# Supplementary figures and images for: An Rtn4/Nogo-A-interacting micropeptide modulates synaptic plasticity with age
Source: PLoS One. 2022 Jun 30;17(6):e0269404. doi: 10.1371/journal.pone.0269404 (PMC9246188; doi:10.1371/journal.pone.0269404)

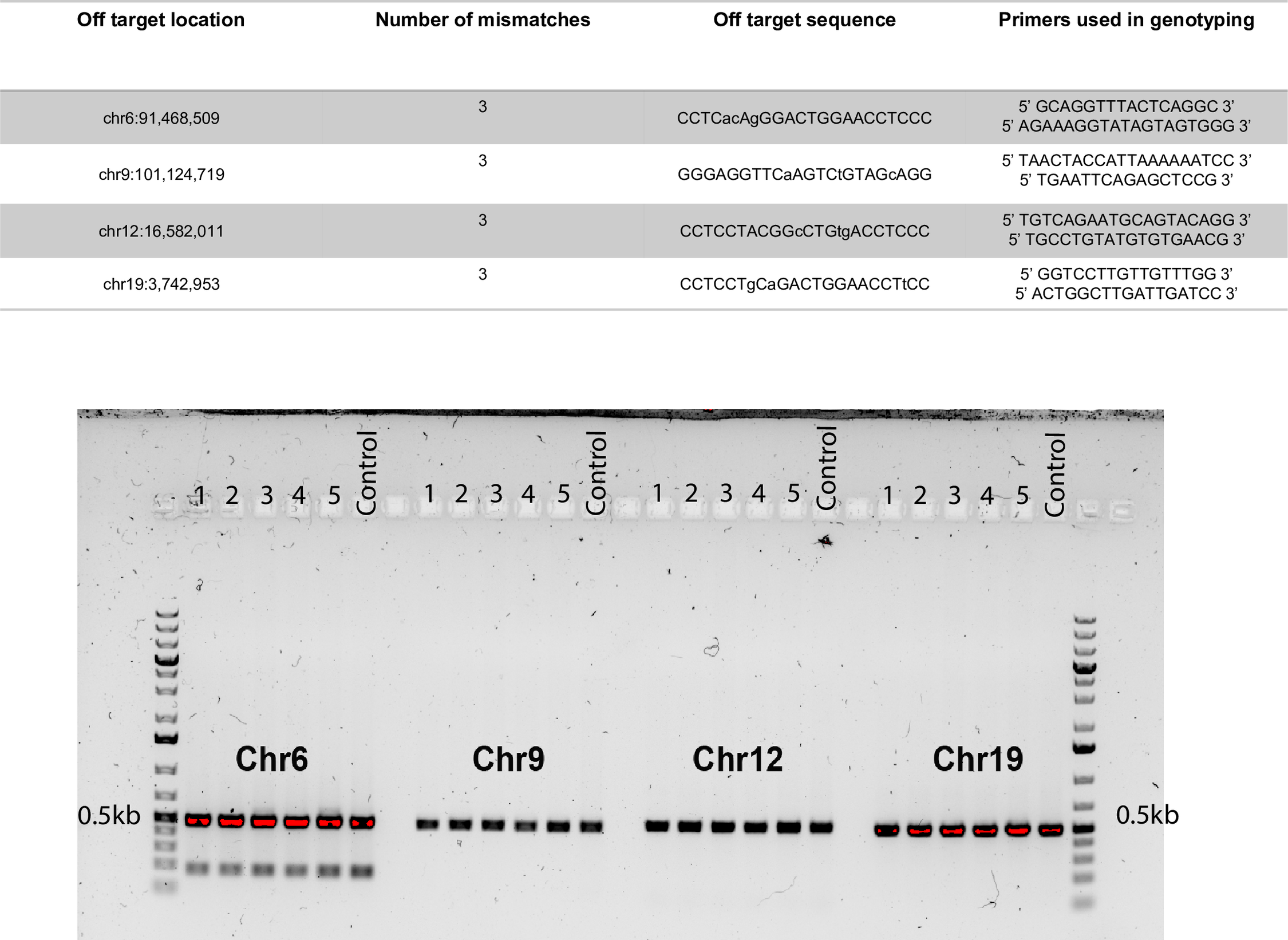

Supplement: S1 Fig — Above, Predicted potential off-target genes for our CRISPR HDR strategy. Primers were designed up and downstream of the target site to produce a 0.5Kb band for detection of the Wildtype locus. PCR was performed using these primer sets from genomic DNA of all founders. Off-targeting would be Indicated by a new band at 850bp, indicating insertion of the tags in the off-target locus. No off-targeting was detected by this method (below). (TIF) [file pone.0269404.s001.tif]

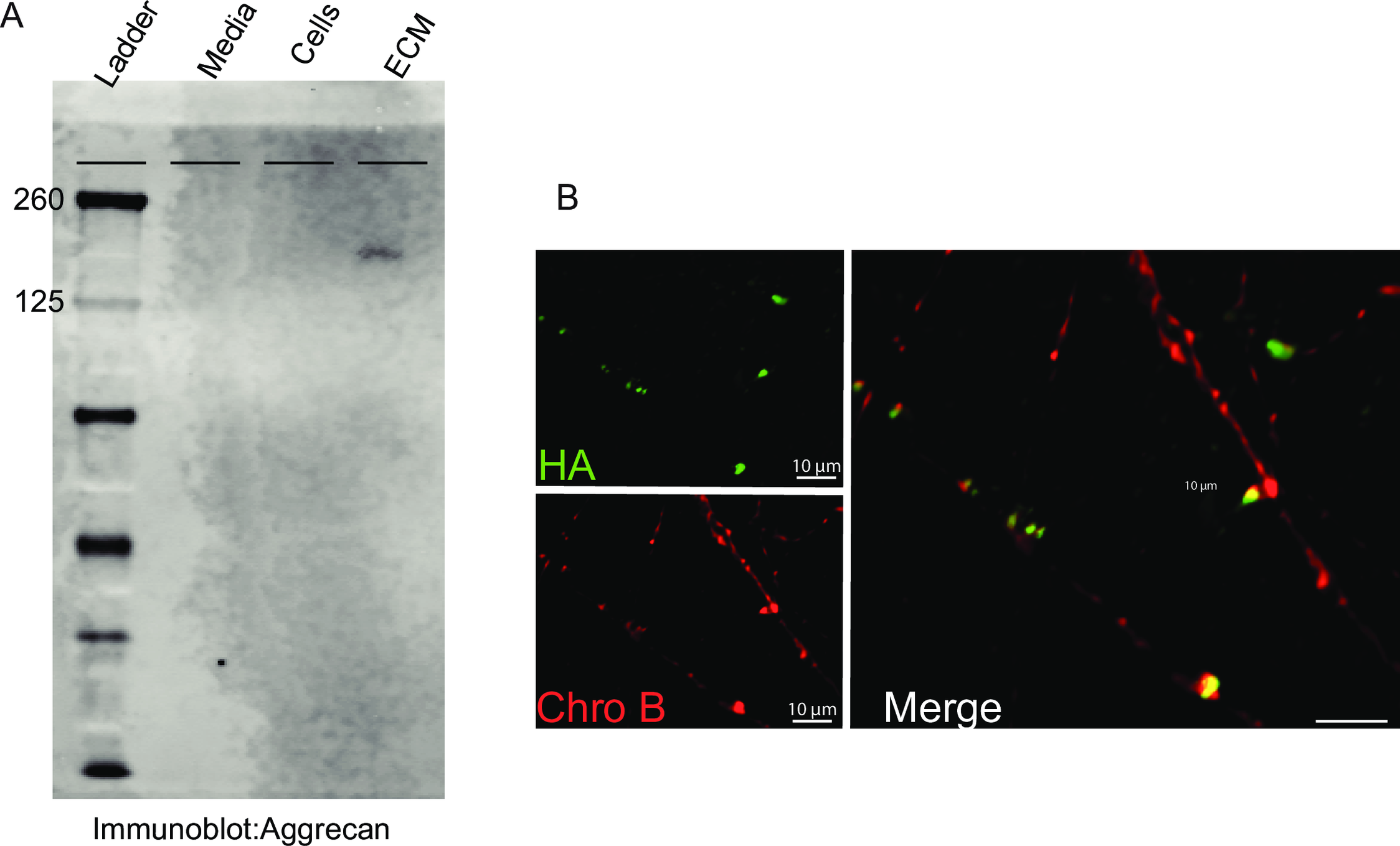

Supplement: S2 Fig — A) The ECM marker protein Aggrecan is found in the ECM fraction, but absent from cell lysate and media protein fractions. B) Immunocytochemistry of PantsSOG-Y primary neurons co-stained with antibodies to HA and the LDCV marker chromogranin B. (TIF) [file pone.0269404.s002.tif]

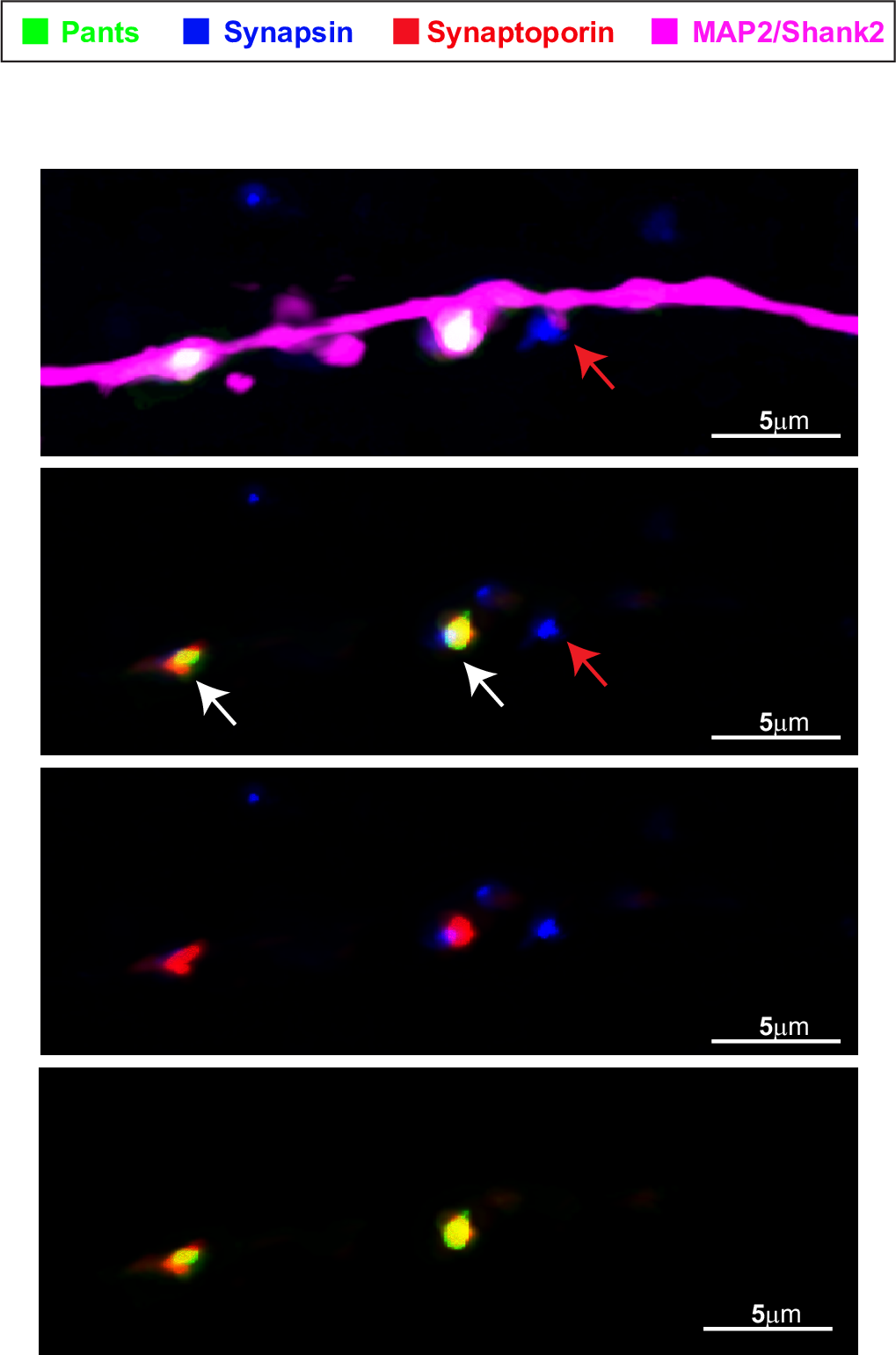

Supplement: S3 Fig — Two morphologically complex spines (white arrows) are positive for Pants (green) and for the TE marker synaptoporin (red), while a mushroom spine (red arrow) is not. (TIF) [file pone.0269404.s003.tif]

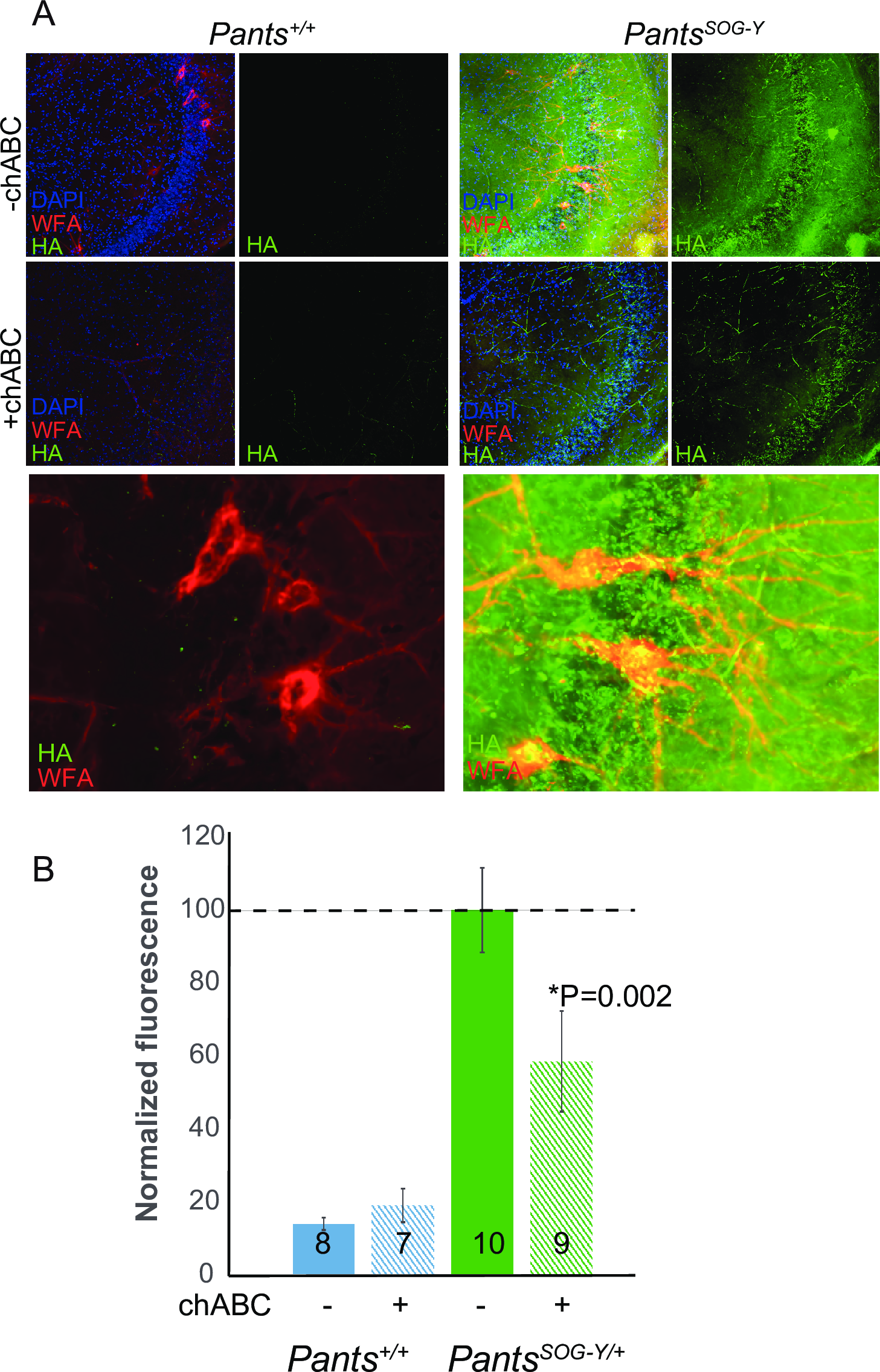

Supplement: S4 Fig — A) Immunohistochemical co-localization of HA with WFA, an ECM marker in 16 week hippocampal area CA3 in the absence and presence of chondroitinase ABC, and enzyme that digests ECM. B) Quantification of fluorescence intensity of HA staining in area CA3 in the absence and presence of chABC (n = 3 mice each condition; number of slices on each bar). (TIF) [file pone.0269404.s004.tif]

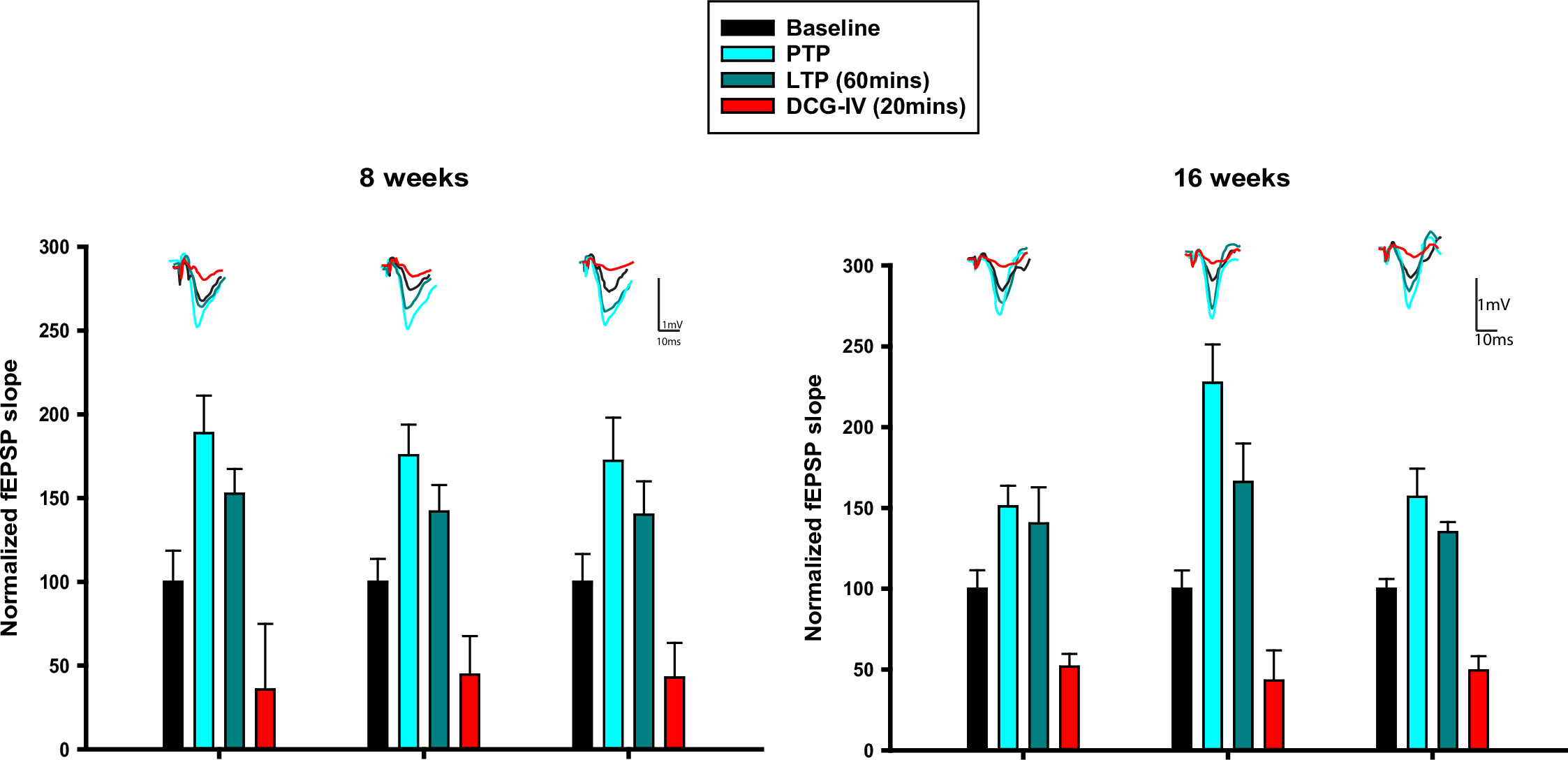

Supplement: S5 Fig — Average slope of field responses at baseline and post-tetanic potentiation and LTP for all 3 genotypes. Red bars indicate field responses after DCG-IV wash-in. Abolishment of LTP by this blocker is a specific feature of MF LTP. (TIF) [file pone.0269404.s005.tif]
