# Supplementary material for: An Rtn4/Nogo-A-interacting micropeptide modulates synaptic plasticity with age
Source: PLoS One. 2022 Jun 30;17(6):e0269404. doi: 10.1371/journal.pone.0269404 (PMC9246188; doi:10.1371/journal.pone.0269404)
Supplement: S1 Raw images — (PDF) [file pone.0269404.s010.pdf]

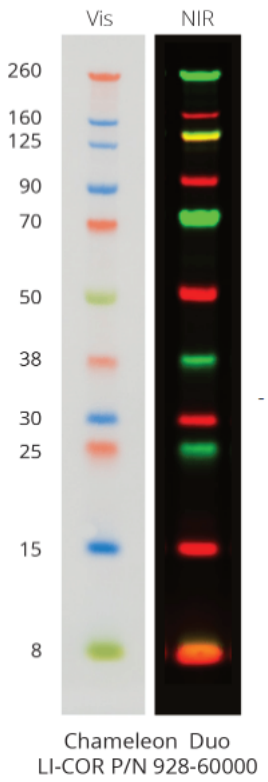

All blots are stained with Odyssey (Li-COR) secondaries in either 680 (red) or 800 (green) unless HRP was used. In both cases, the Chameleon Duo ladder was used, which is visible under white light and also has band that fluoresc in both channels (key left). I have labeled each blot as simply ‘red’ or ‘green’ so that the sizes of the ladder bands can be discerned. All blots were captured with the Odyssey imaging system, except for HRP blots, which were captured with the ECL setting of a ChemiDoc imager (Bio-Rad).

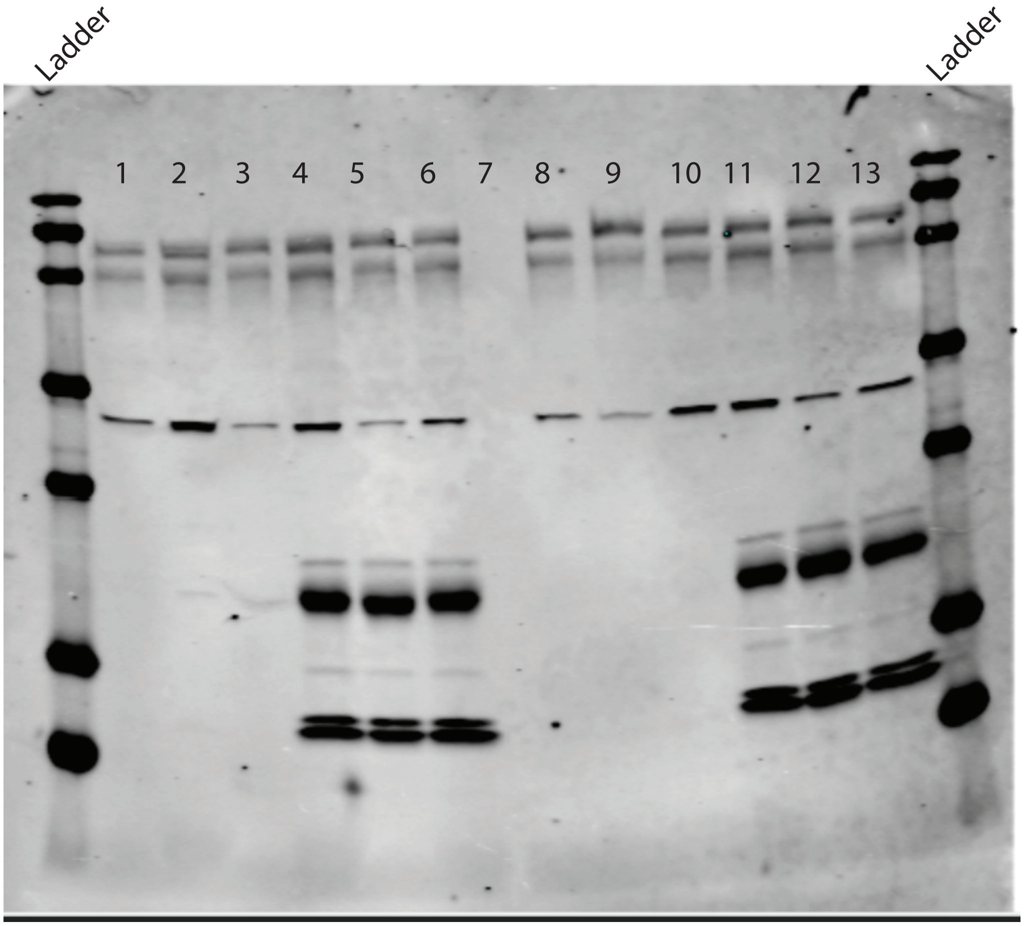

Figure 1C Top panel-HA blot (red channel) in hippocampal extracts IP’d for HA-Pants. Lane 7 is empty. Each lane represents hippocampus extracts from a different animal. Lanes 1-6 are 8 week. Lanes 8-13 are 16 week. Lanes 1-3 and 8-10 are WT (nontransgenic) controls. Lanes 4-6 and 11-13 are *Pants*$^{SOG/SOG}$.

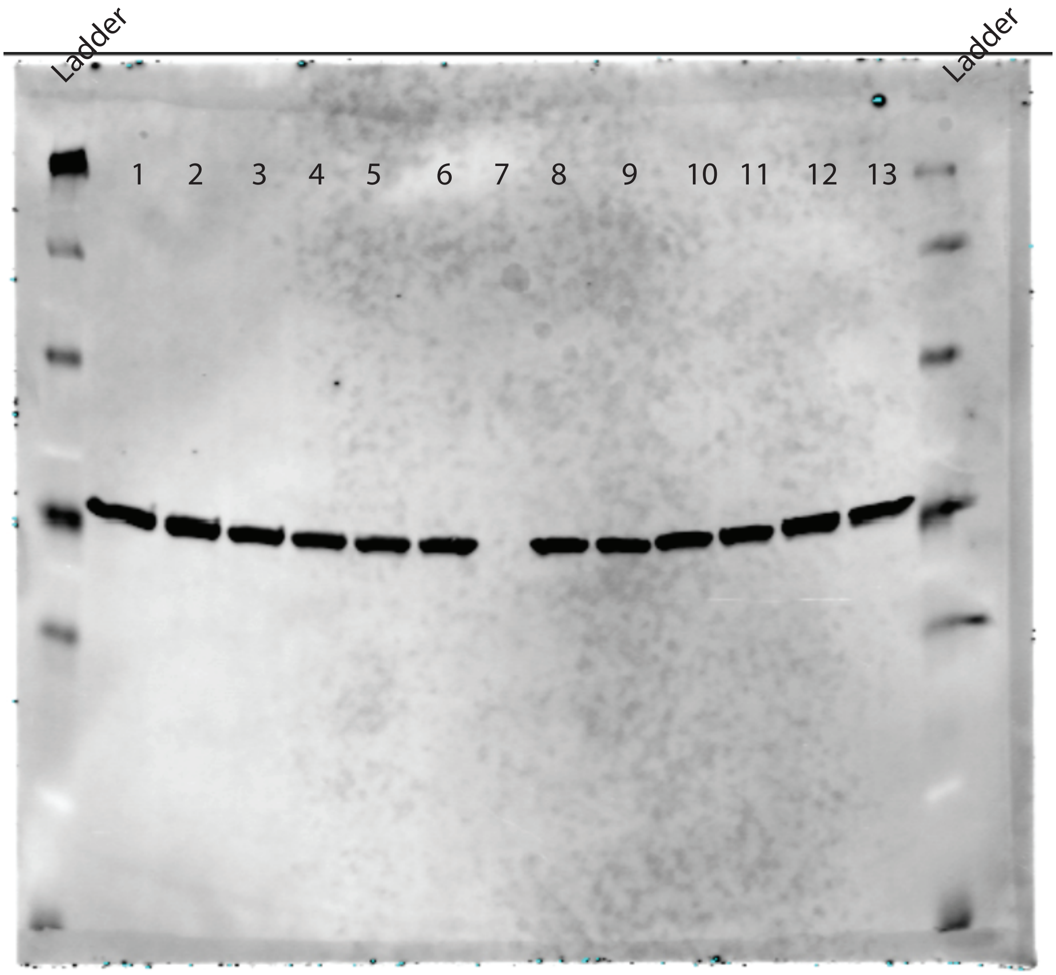

Figure 1C Bottom panel-B-Actin (green channel) in hippocampal extracts. Lane 7 is empty. Each lane represents hippocampus extracts from a different animal. Lanes 1-6 are 8 week. Lanes 8-13 are 16 week. Lanes 1-3 and 8-10 are WT (nontransgenic) controls. Lanes 4-6 and 11-13 are *Pants*$^{SOG/SOG}$.

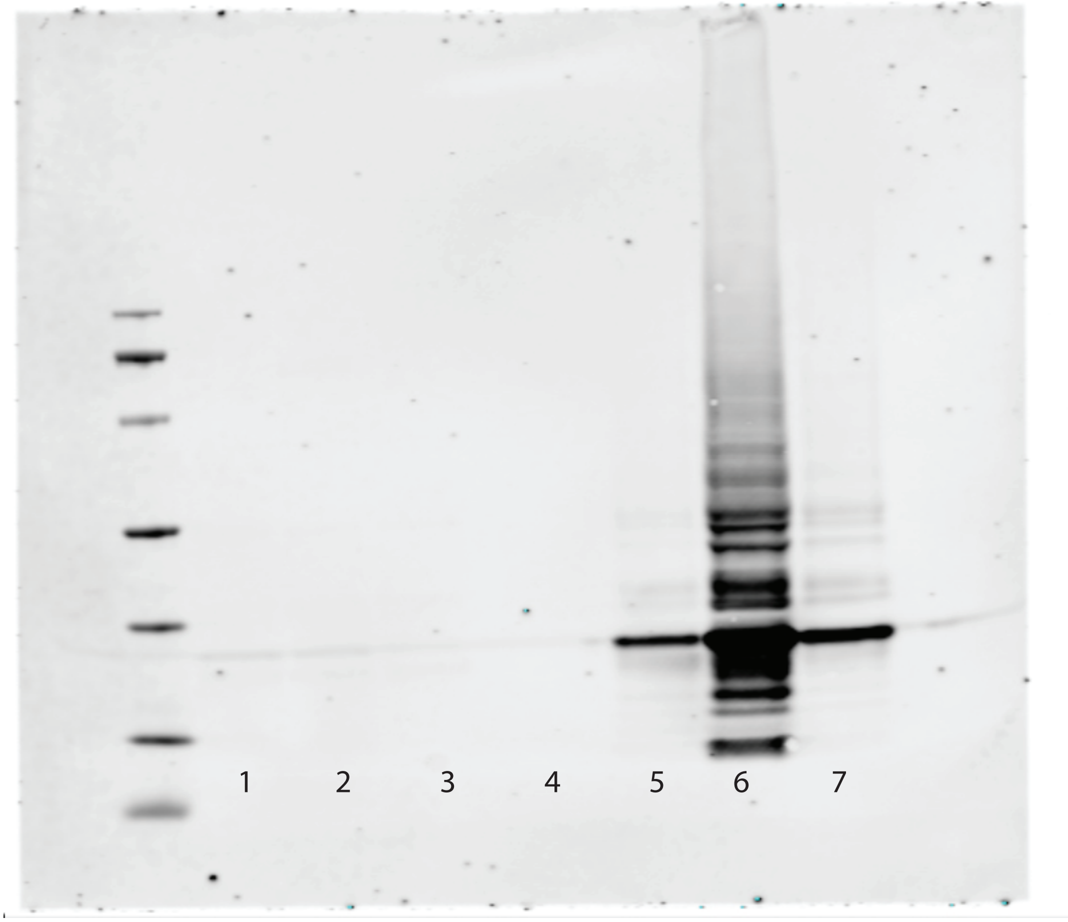

Figure 2D HA (red channel) in Cell lysates, media, or ECM scrapes. Lane 4 is empty. Lanes 1-3 are untransfected controls. Lanes 5-7 were transfected with Pants-SOG-HA overexpression construct

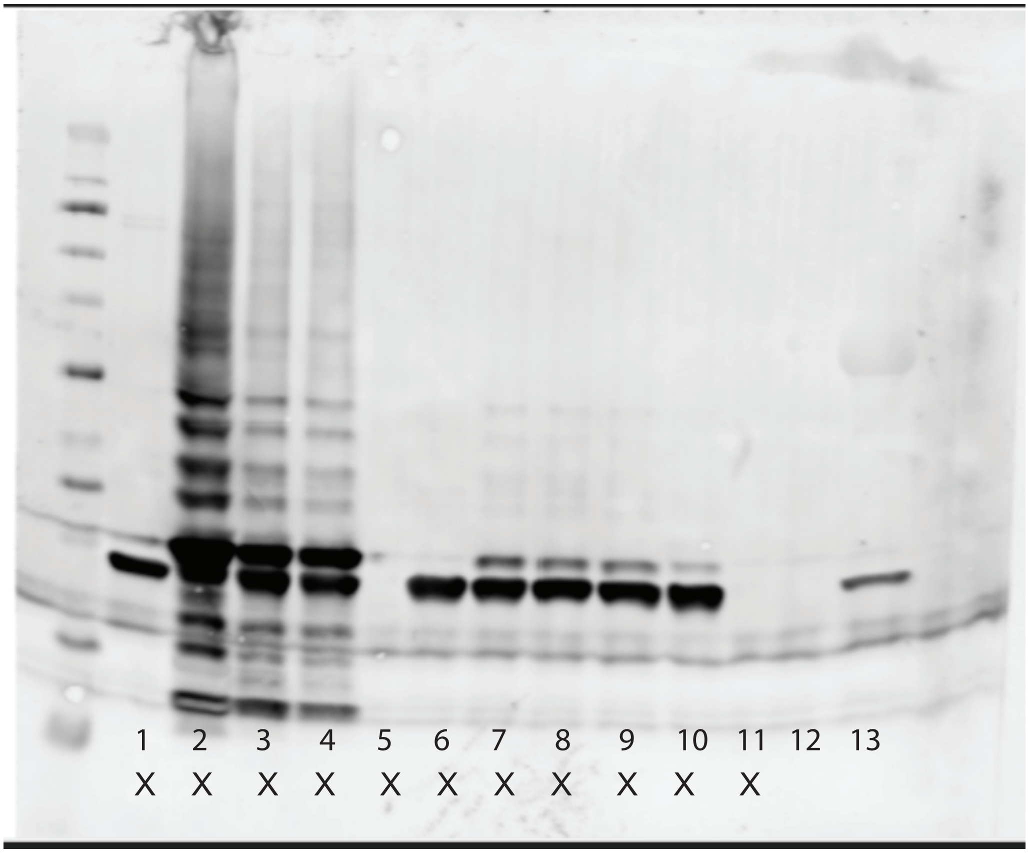

Figure 2E HA (red channel) immunoblot of media from PantsSOGY primary hippocampal neuron cultures. Lanes 1-11 are from an unrelated experiment. Lane 12 is media from Wildtype (nontransgenic control) neurons and Lane 13 is media from transgenic neuron cultures

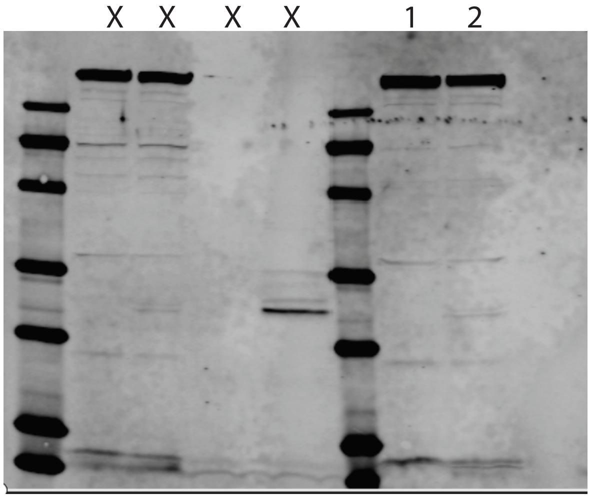

Figure 5C V5 IB of N2A cell lysates (red channel)
Top panel. Lane 1 is WT control cells (no knockin
of V5-TurboID-t2AGFP and Lane 2 is the knockin cell line

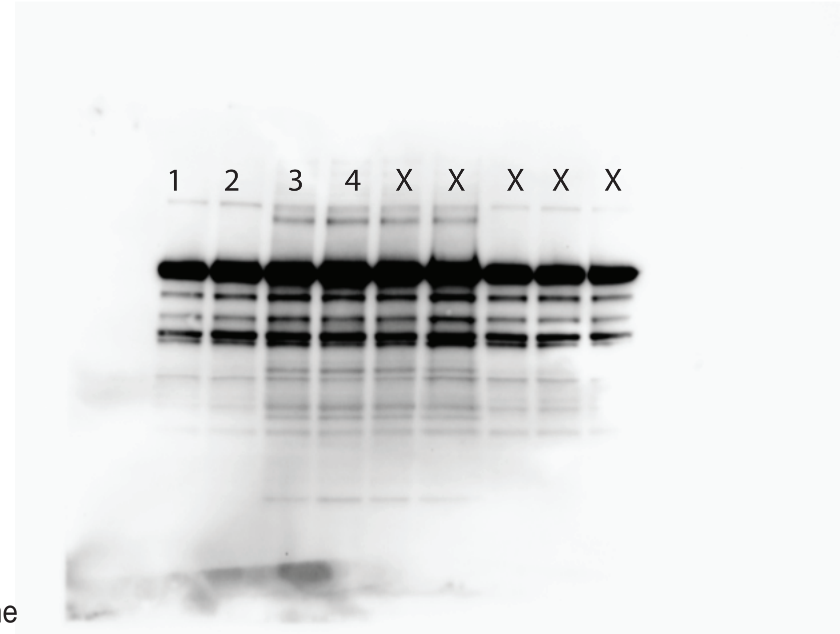

Figure 5D IB: Streptavidin-HRP followed by ECl and Chmi-doc imaging. Cells were labeled with Biotin (50mcm 18 hours), lysates were prepared and WB run on lysates. Lanes 1 and 2 are WT controls (no TurboID). Lanes 3-4 are Pants-TurboID cell lines. Below is a colorimetric image of the ladder taken without moving the blot to align the ladder.

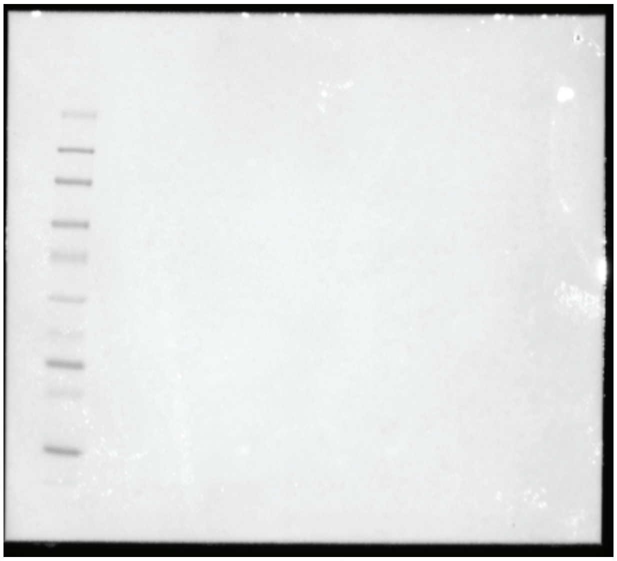

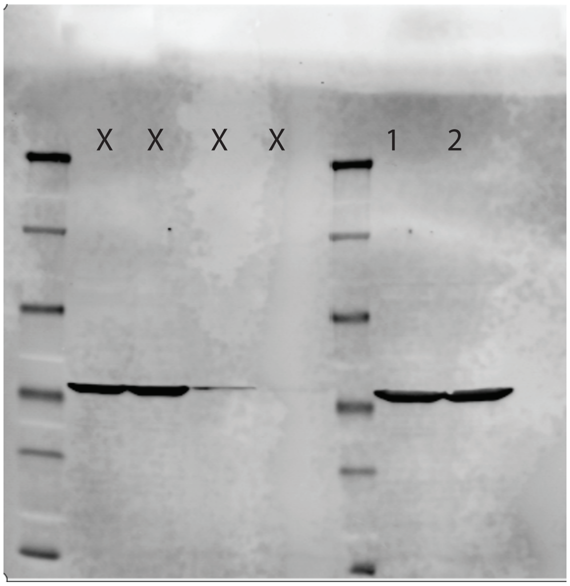

Figure 5C Beta Actin IB of N2A cell lysates (Green channel)
Bottom panel. Lane 1 is WT control cells (no knockin
of V5-TurboID-t2AGFP and Lane 2 is the knockin cell line

For figure 5H, lanes labeled 1 and 2 represents lysates from biotinylated cells that are WT (no TurboID transgene) controls (lane 1) or Tg cell line, where TurboID is fused to the 3'end of the Pants gene. Comparison of lanes 1 and 2 shows equivalent expression of the gene of interest. Lanes 3 and 4 are eluates from streptavidin pulldown of biotinylated proteins from WT (lane 3) or TurboID transgenic cell line (lane 4)

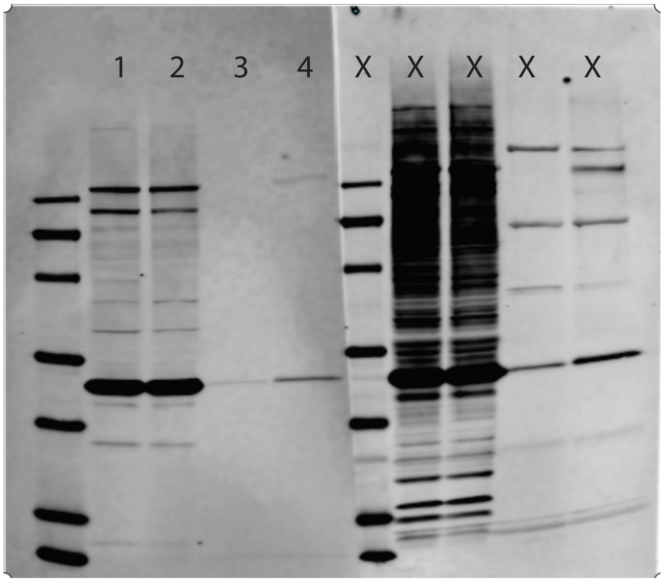

Figure 5H Nogo-A IB (red channel) Top panel

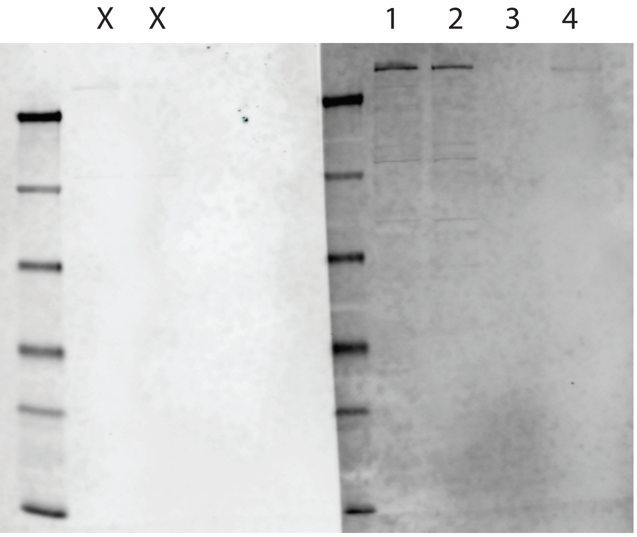

Figure 5H RanBP2 (green channel) 4th panel

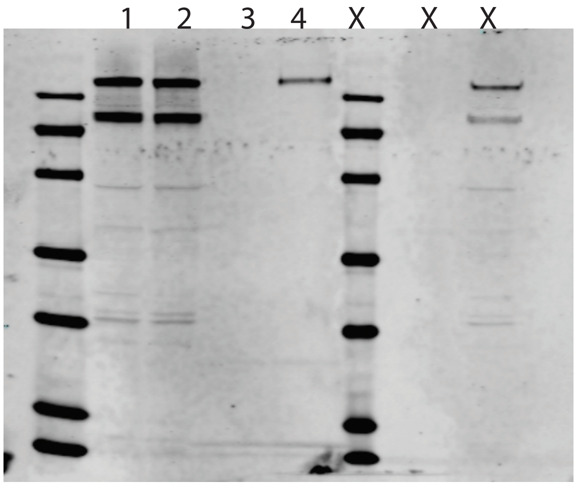

Figure 5H NCam-1 IB (red channel) 2nd panel

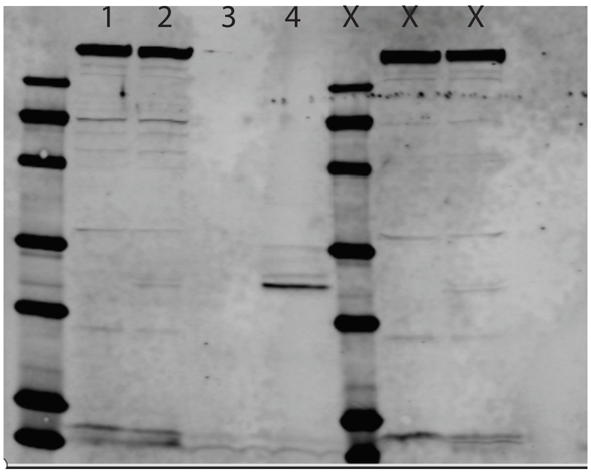

Figure 5H V5 (red channel) 5th panel

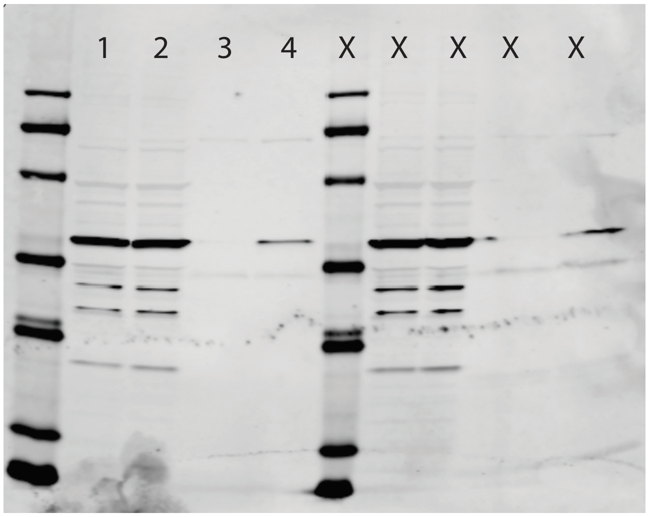

Figure 5H CCT IB (red channel) 3rd panel

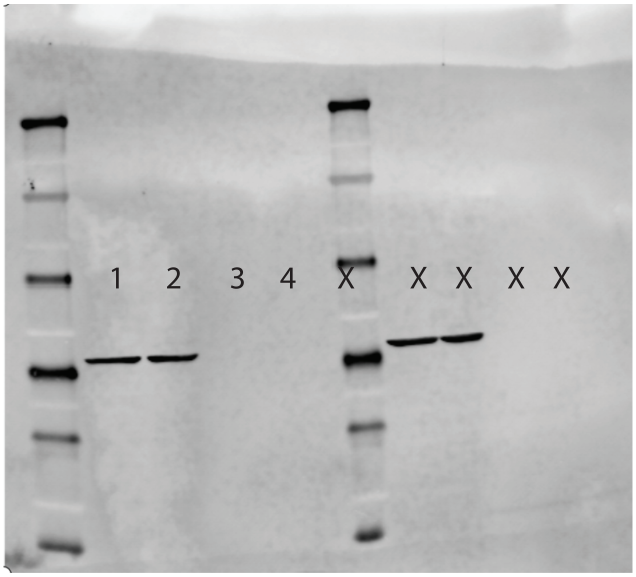

Figure 5H Beta Actin (Green channel) 6th panel

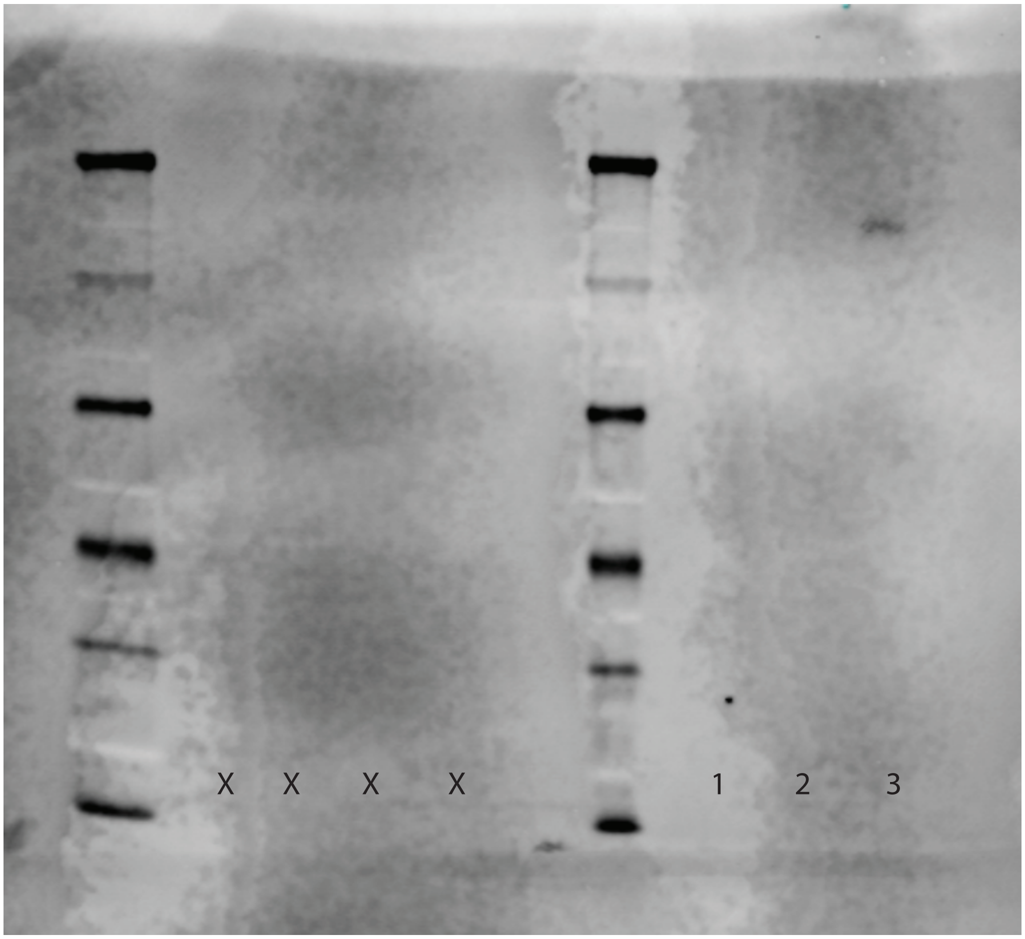

Figure S2A IB: Aggrecan (green channel) Cell lysates (lane 1), media(Lane 2), or ECM scrapes (lane 3) from N2A cultures transfected with Pants-SOG-HA overexpression construct

All gels are of PCR products as described in the text. Gels are imaged with SYBR safe on a Chemi-doc imager (Bio-Rad) using automatic detection settings. Ladders are the ThermoFisher 1Kb Plus ladder, shown below

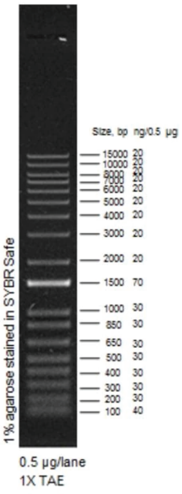

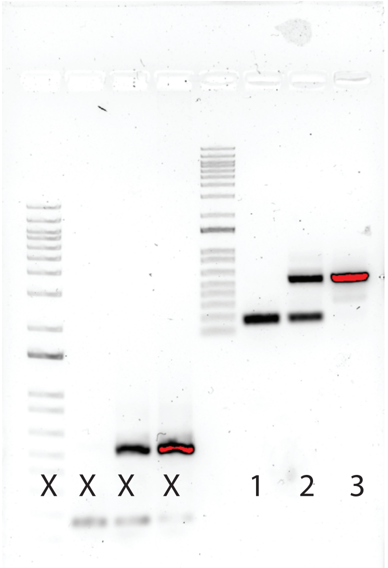

Fig 1B genotyping of novel PantsSOG Transgenic mouse line for 1 WT(lane 1), 1Het (Lane 2) and 1 homozygous Knockin (lane 3) animal.

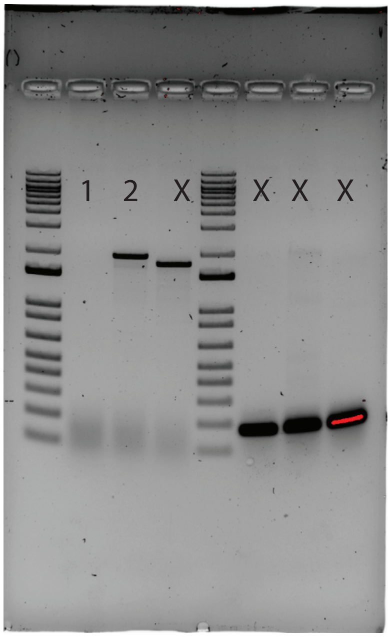

Fig 5B genotyping of novel Pants-TurboID Transgenic cell line for 1 WT(lane 1), Knock-in line (Lane 2)

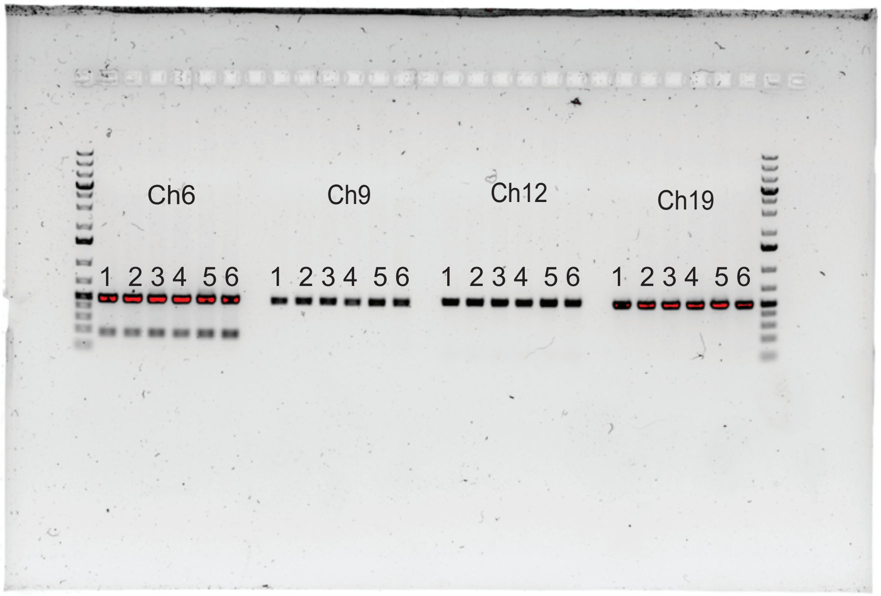

Fig S1B PCR testing for off-target knockin of the SOG-Y targeting sequence at all predicted sites. Numbers 2-6 represent genomic DNA from a different founder. Number 1 is a WT control. The site being tested is listed above each group.
